# A causal relationship between hypothyroidism and rheumatoid arthritis, but not hyperthyroidism: evidence from the mendelian randomization study

**DOI:** 10.1007/s00508-024-02386-6

**Published:** 2024-06-20

**Authors:** Mingyi Yang, Yani Su, Ke Xu, Pengfei Wen, Jianbin Guo, Zhi Yang, Lin Liu, Peng Xu

**Affiliations:** Department of Joint Surgery, HongHui Hospital, Xi’an Jiaotong University, 710054 Xi’an, Shaanxi China

**Keywords:** Hyperthyroidism, Hypothyroidism, Rheumatoid arthritis, Single nucleotide polymorphism, Causal

## Abstract

**Objective:**

To investigate the genetic level causal association among hyperthyroidism, hypothyroidism, and rheumatoid arthritis (RA).

**Methods:**

We utilized the genome-wide association studies (GWAS) summary data for exposure (hyperthyroidism and hypothyroidism) and outcome (RA) from the IEU OpenGWAS database. We used two different sets of data (test cohort and validation cohort) for causal assessment of exposure and outcome. To establish a causal relationship between these conditions, we conducted a two-sample Mendelian randomization (MR) analysis. Subsequently, we evaluated the MR analysis results for heterogeneity, horizontal pleiotropy, and outliers, aiming to assess the validity and reliability of the findings. Moreover, we conducted additional analyses to examine the robustness of the MR results, including a “Leave one out” analysis and the MR robust adjusted profile score (MR-RAPS) method, ensuring the robustness and adherence to normal distribution assumptions.

**Results:**

The findings from the test cohort indicated that hyperthyroidism did not exhibit a genetic causal association with RA (*P* = 0.702, odds ratio [OR] 95% confidence interval [CI] = 1.021 [0.918–1.135]). Conversely, hypothyroidism displayed a positive genetic causal relationship with RA (*P* < 0.001, OR 95% CI = 1.239 [1.140–1.347]). The analysis results of the validation cohort are consistent with those of the test cohort. Notably, our MR analysis results demonstrated no evidence of heterogeneity, horizontal pleiotropy, or outliers. Furthermore, our MR analysis results remained unaffected by any single nucleotide polymorphism (SNP) and exhibited a normal distribution.

**Conclusion:**

The results of this study showed that hypothyroidism was positively correlated with RA, while hyperthyroidism was not causally correlated with RA. Hypothyroidism may as a risk factor of RA should be paid attention to in clinical work. Future studies are needed to further confirm this finding.

**Supplementary Information:**

The online version of this article (10.1007/s00508-024-02386-6) contains supplementary material, which is available to authorized users.

## Introduction

Rheumatoid arthritis (RA) is a chronic systemic autoimmune disease characterized by symmetrical multiarticular synovitis. Inflammatory cell infiltration results in the degeneration of synovitis, articular cartilage, and bone [1, 2]. RA primarily exhibits chronic, symmetrical, multisynovial arthritis and extra-articular lesions, predominantly affecting small joints such as the hands, wrists, and feet, with recurrent and symmetrical symptoms [3]. RA patients may experience manifestations in other organs, including interstitial lung disease, pericarditis, pleural effusion, or bronchiectasis [3]. The incidence of RA ranges from 0.5 to 1%, with a male-to-female ratio of 2.5:1. Although RA can occur at any age, it most commonly emerges between 40 and 70 years old. The age of onset is positively associated with the incidence of RA, and approximately 40% of RA patients become disabled within 10 years [4]. Presently, genetic and environmental factors are recognized as the primary regulatory factors for RA [5]. Individuals with a positive family history have a 3- to 5‑fold increased risk of developing RA [6]. The heritability of serological positive RA is estimated to be 40–65%, whereas serological negative RA has a heritability of 20% [7, 8]. Currently, there is no cure for RA. In advanced stages of the disease, surgical interventions, such as total knee replacement and total hip replacement, are necessary for large joint management. The chronic joint inflammation associated with RA leads to joint damage, functional loss, and various musculoskeletal impairments, ultimately resulting in decreased physical and social functioning in patients. Moreover, advanced RA not only causes joint destruction and disability but also contributes to multiple secondary complications, including cardiovascular disease, significantly impacting both the quality of life for patients and society as a whole.

Hyperthyroidism is a clinical syndrome characterized by an excessive production and secretion of thyroid hormones by the thyroid gland, resulting in a state of hypermetabolism. This condition is distinguished by elevated levels of free serum thyroxine (T4) and/or free triiodothyronine (T3) [9]. Hyperthyroidism is a prevalent disorder worldwide, with a global prevalence ranging from 0.2–1.3% [10]. In the United States, the overall prevalence of hyperthyroidism is reported as 1.2%, with dominant hyperthyroidism accounting for 0.5% and subclinical hyperthyroidism accounting for 0.7% [11]. The primary causes of hyperthyroidism are commonly attributed to Graves’ disease (GD), toxic multinodular goiters, and toxic adenomas [11]. Clinical manifestations of hyperthyroidism encompass a range of symptoms, including increased heat production and basal metabolic rate due to cellular effects mediated by T3-binding receptors, resulting in weight loss and fatigue. Additionally, individuals with GD may exhibit skin changes such as thinning hair and anterior tibial mucous edema [11]. Hyperthyroidism can significantly impact various systems in the body, including the integumentary, musculoskeletal, immune, ophthalmic, reproductive, gastrointestinal, and cardiovascular systems, posing a substantial threat to overall human health [11]. Hypothyroidism, on the other hand, is a frequently encountered clinical disorder and the most prevalent hormonal deficiency disorder. It occurs when the thyroid gland fails to produce adequate amounts of thyroid hormone to meet the demands of surrounding tissues [12]. In its early stages, hypothyroidism often remains latent and may exhibit no noticeable symptoms. However, as the disease progresses, nonspecific symptoms begin to manifest, commonly including fatigue, cold tolerance, and constipation [13]. Thus, the clinical presentation of hypothyroidism can vary significantly [14]. Hypothyroidism ranges in severity from asymptomatic cases to severe conditions leading to coma and multiple organ failure [15]. It is more commonly observed in women, with the incidence increasing with age [15]. Overt hypothyroidism has a prevalence ranging from 0.3–3.7% in the United States and from 0.2–5.3% in Europe. Subclinical hypothyroidism exhibits an even higher prevalence, estimated to be between 3–15% [12]. If left untreated, severe hypothyroidism can result in heart failure, psychosis, and coma, thus imposing considerable distress on individuals and society at large [15].

**Fig. 1:**
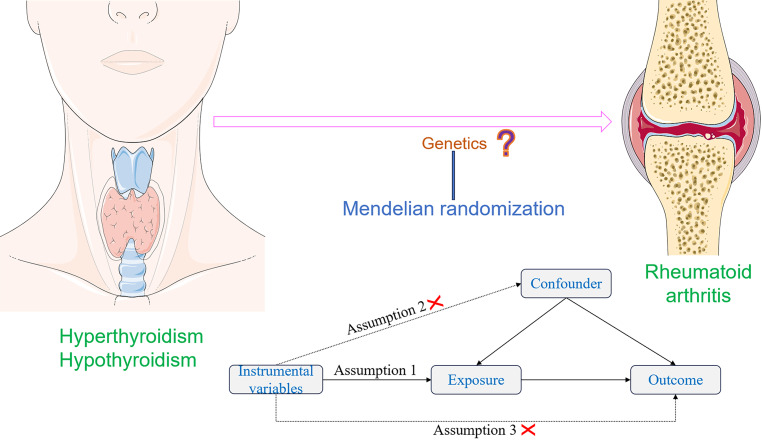
The graphical abstract of this study. The genetic causal relationship between hyperthyroidism, hypothyroidism and rheumatoid arthritis was investigated by MR analysis. Follow three basic assumptions: firstly, the instrumental variables should exhibit a strong association with the exposure. Secondly, the instrumental variables utilized must be independent of both the outcome and any potential confounding factors. Lastly, instrumental variables affect outcomes only through exposure

Genome-wide association studies (GWAS) have significantly advanced the field of human disease genetics, and the publicly available GWAS summary data provides a vast database for further genetic investigations into various diseases and traits [16]. Mendelian randomization (MR) is a statistical approach employed for inferring epidemiological etiology analysis, following Mendel’s law of heredity, which entails the random assignment of alleles. Utilizing extensive GWAS summary data, MR analysis utilizes single nucleotide polymorphisms (SNPs) as instrumental variables (IVs) to investigate genetic causality between exposure and outcome [17]. MR effectively controls for confounding factors by leveraging the innate nature of genetic mutations, which remain unaffected by environmental influences. Furthermore, as genetic variations can influence outcomes, but outcomes cannot impact genes, MR avoid the issue of reverse causation [18]. Previous studies have identified a causal relationship between hyperthyroidism and venous thromboembolism through MR analysis [19], as well as a causal link between hypothyroidism and systemic lupus erythematosus [17]. In this study, MR analysis was employed to examine the potential causal association between hyperthyroidism, hypothyroidism, and RA. The graphical abstract of this study is shown in Fig. 1.

## Materials and methods

### Data source

The GWAS summary data pertaining to exposure (hyperthyroidism and hypothyroidism) and outcome (RA) were obtained from the IEU OpenGWAS database. Two distinct datasets were utilized: the test cohort and the validation cohort. The exposure (hyperthyroidism and hypothyroidism) of test cohort were obtained from the IEU OpenGWAS database. The outcome (RA) of test cohort were obtained from the Finnish consortium. The exposure (hyperthyroidism and hypothyroidism) of validation cohort were obtained from the Finnish consortium. The outcome (RA) of validation cohort were obtained from the UK Biobank. Within the test cohort, the GWAS summary data for hyperthyroidism comprised 460,499 individuals (3557 cases and 456,942 controls), encompassing 24,189,279 SNPs. Similarly, the GWAS summary data for hypothyroidism in the test cohort involved 410,141 individuals (30,155 cases and 379,986 controls), with 24,138,872 SNPs identified. Additionally, the GWAS summary data for RA within the test cohort included 153,457 individuals (6236 cases and 147,221 controls), capturing 16,380,169 SNPs. Within the validation cohort, the dataset of hyperthyroidis comprised a sample of 173,938 individuals, with 962 cases and 172,976 controls. A total of 16,380,189 SNPs were identified in this dataset. Similarly, the dataset of hypothyroidism for validation cohort included 198,472 individuals, with 22,997 cases and 175,475 controls. This dataset produced 16,380,353 SNPs. For the outcome data of validation cohort, the GWAS summary data for RA consisted of 361,194 individuals, with 1605 cases and 359,589 controls. A total of 10,079,899 SNPs were identified in this dataset. All populations in this study were of European descent and included both males and females. It is worth noting that all the GWAS summary data employed in this study were obtained from publicly available databases; hence, no informed consent or ethical statements were required. **Supplementary Table 1** provides additional details regarding the data employed in this study.

### IVs selection

The validity of MR analysis relies on the satisfaction of three fundamental assumptions. Firstly, the IVs employed in MR analysis should exhibit a strong association with the exposure of interest. Secondly, the IVs utilized must be independent of both the outcome under investigation and any potential confounding factors. Lastly, the IVs should exclusively impact the outcome through their influence on the exposure. To ensure the robustness of the genetic causal assessment of exposure to outcome, we subjected the IVs to a rigorous screening process encompassing several quality control measures. Initially, we evaluated the strength of the association between the IVs and the exposure, requiring a significance level of *P* < 5 × 10^−8^ and an F statistic > 10. The F statistic was calculated using the formula: F = R^2^(N-K-1) / K(1‑R^2^). Subsequently, we accounted for the potential bias arising from linkage disequilibrium (LD) among the SNPs closely associated with the exposure. To address this concern, we established specific conditions for LD, necessitating an r^2^ < 0.001 and clump distance > 10,000 kb. Moreover, we excluded any SNPs associated with the outcome at a significance level of *P* < 5 × 10^−8^ from the pool of IVs. Additionally, we leveraged the PhenoScanner database (http://www.phenoscanner.medschl.cam.ac.uk/phenoscanner) to identify and exclude SNPs linked to confounding factors. Specifically, smoking and obesity, recognized risk factors for RA, were identified as confounders and excluded from the genetic causal assessment between exposure and outcome. Furthermore, we implemented two additional exclusion criteria for the selection of IVs. First, we eliminated palindromic SNPs with intermediate allele frequencies, and second, we excluded SNPs exhibiting incompatible alleles from the pool of IVs. Lastly, in cases where the SNPs failed to match the GWAS summary data for the outcome, we employed the LDlink online platform to obtain proxy SNPs.

### Statistical analysis

We conducted a two-sample MR analysis using the “TwoSampleMR” and “MendelianRandomization” packages in R (version 4.1.2). Five different MR analysis methods were employed to examine the genetic causality between exposure and outcome: random-effects inverse variance weighted (IVW), MR Egger, weighted median, simple mode, and weighted mode. The primary analysis method used was random-effects IVW, which determines the genetic causality between exposure and outcome based on its results. The IVW method is a well-established and widely used statistical approach in MR, assuming the validity of all included SNPs as instrumental variables. It calculates the Wald ratio for each SNP and combines them to evaluate the causal effect of exposure on outcome. In the absence of horizontal pleiotropy, IVW offers greater statistical power and provides a reliable assessment of causality between exposure and outcome [20]. MR-Egger regression, on the other hand, can detect pleiotropy through an intercept term and provide causal estimates after adjusting for pleiotropic effects. However, this method compromises statistical power [21]. The weighted median approach assumes that at least half of the instrumental variables are valid and can provide a causal assessment of exposure and outcome [22]. The simple mode is a model-based estimation method that ensures robustness against pleiotropy [23]. The weighted mode method offers causal estimates when the majority of similar individual estimates are derived from valid instrumental variables [24]. Nonetheless, compared to the IVW method, MR Egger, weighted median, simple mode, and weighted mode exhibit lower statistical power. Therefore, in cases where the analysis results of random-effects IVW are inconsistent with the other four methods, we still consider the results from random-effects IVW [24].

For the analysis of exposure and outcome in the context of MR, several tests were conducted to assess the reliability of the causal evaluation. Firstly, heterogeneity was examined using Cochran’s Q statistic for MR-IVW and Rucker’s Q statistic for MR Egger. A *p*-value > 0.05 indicated a lack of heterogeneity. Secondly, the presence of horizontal pleiotropy was assessed through the intercept test of MR Egger and the MR pleiotropy residual sum and outlier (MR-PRESSO). A *p*-value > 0.05 suggested the absence of horizontal pleiotropy. Thirdly, using the MR-PRESSO to investigate whether the causal evaluation was influenced by outliers. Fourthly, a “Leave one out” analysis was performed by assessing the causality of one SNP remaining after each removal of other SNPs. This analysis aimed to determine whether the genetic causality assessment was impacted by any single SNP. Fifthly, the robustness of the findings was evaluated using the MR robust adjusted profile score (MR-RAPS) method, which examined whether the genetic causality assessment between exposures and outcome adhered to a normal distribution. A *p*-value > 0.05 indicated conformity to the normal distribution.

## Results

### Test cohort

#### IVs selection

After excluding LD, a total of 13 SNPs exhibited significant correlation with hyperthyroidism, while 75 SNPs showed strong correlation with hypothyroidism. Among them, 12 SNPs were chosen as alternative IVs for conducting MR analysis of hyperthyroidism and RA. Three SNPs associated with the outcome (rs6679677, rs2856821 and rs3087243) were excluded, no confounding SNPs could be detected. Additionally, one palindromic SNP (rs385863) was removed. Consequently, a set of eight SNPs was obtained as IVs for the causal evaluation of hyperthyroidism and RA, as presented in **Supplementary Table 2**. For the analysis of hypothyroidism and RA, a total of 70 SNPs were selected as alternative IVs. Eight SNPs (rs9271365, rs6679677, rs9277559, rs9264277, rs3087243, rs7574865, rs3184504 and rs34536443) associated with the outcome were excluded, along with one confounding SNPs (rs4409785), and two palindromic SNPs (rs2412976 and rs2921053). In addition, 11 outliers (rs11171710, rs12379417, rs2988277, rs3118469, rs11875260, rs12117927, rs1364450, rs244685, rs7441808, rs2247314 and rs7030280) were removed through MR-PRESSO analysis. Finally, a set of 48 SNPs was obtained as IVs for the causal evaluation of hypothyroidism and RA, as presented in **Supplementary Table 3**.

#### MR analysis

**Fig. 2:**
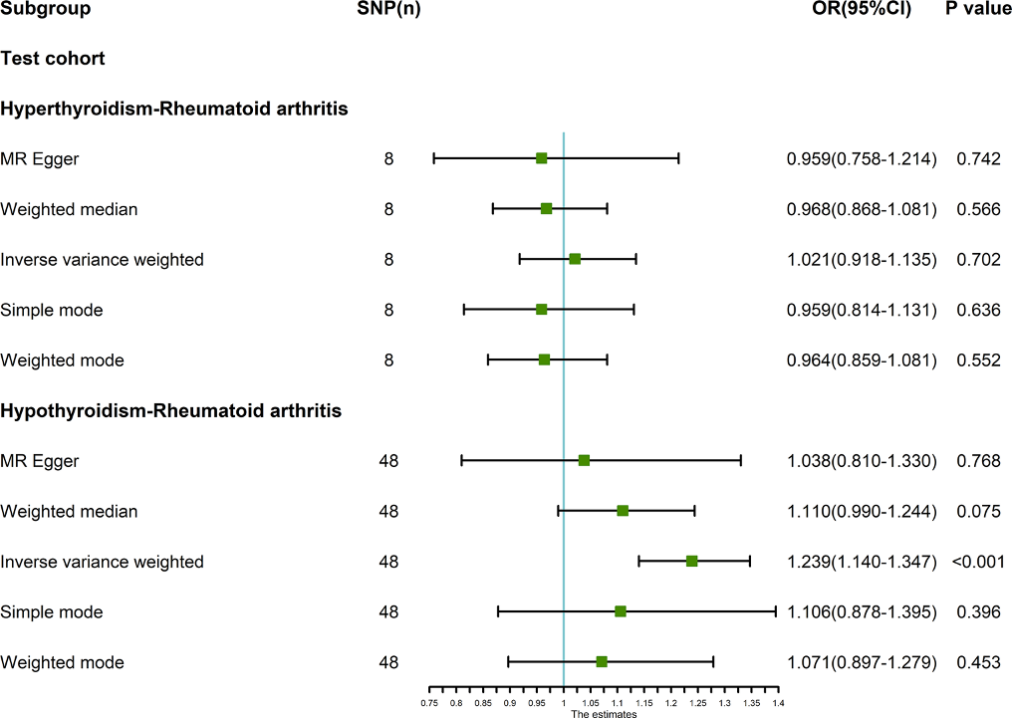
MR analysis results of hyperthyroidism, hypothyroidism and rheumatoid arthritis of test cohort. Five methods: random-effects IVW, MR Egger, weighted median, simple mode, and weighted mode. The primary analysis method used was random-effects IVW. Random-effects IVW is the gold standard of MR analysis, supplemented by MR Egger, weighted median, simple mode, and weighted mode. If the results of the five methods are inconsistent, the random-effects IVW analysis result prevails

**Fig. 3:**
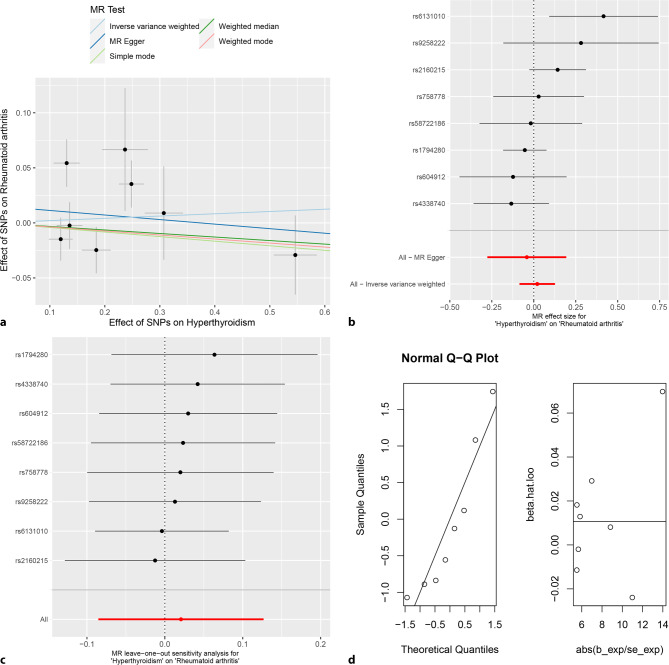
MR analysis of hyperthyroidism and rheumatoid arthritis for test cohort. **a** Scatter plot, the effect of SNPs on hypothyroidism in horizontal coordinate and effect of SNPs on rheumatoid arthritis in vertical coordinate. **b** The forest map, contains a total of 8 SNPs. Each horizontal line segment represents an SNP effect estimate and its 95% confidence interval. All-MR-Egger represents a comprehensive estimate of the effect size of all SNPs using the MR-Egger method. All-IVW represents a comprehensive estimate of the effect size of all SNPs using the IVW method. **c** Leave-one-out analysis, delete other SNPs, and use the remaining SNP for MR analysis. All represents an estimate of the total effect size including all SNPs using the IVW method. Each horizontal line represents an estimate of the effect of all SNPs on the result, with the exception of a particular SNP, and its 95% confidence interval. **d** Normal distribution map, to test whether genetic causality conforms to normal distribution. A total of 8 SNPs were included, and the symmetrical distribution of SNPs on both sides of the oblique line indicated that the genetic causality was normally distributed

The random-effects IVW analysis indicated that there was no genetic causal relationship between hyperthyroidism and RA (*P* = 0.702, odds ratio [OR] 95% confidence interval [CI] = 1.021 [0.918–1.135]). Similar results were observed across multiple methods, including MR Egger, weighted median, simple mode, and weighted mode, which all failed to establish a genetic causal relationship between hyperthyroidism and RA (*P* > 0.05) (Figs. 2, 3a and 3b**)**. Notably, both the Cochran’s Q statistic for MR-IVW and Rucker’s Q statistic for MR Egger demonstrated no heterogeneity in the genetic causality assessment between hyperthyroidism and RA (*P* > 0.05). The intercept test for MR Egger and the MR-PRESSO provided no evidence of horizontal pleiotropy in the genetic causality assessment (*P* > 0.05). Additionally, the MR-PRESSO indicated the absence of any outlier affecting the genetic causality assessment between hyperthyroidism and RA **(**Table 1**)**. The “Leave one out” analysis demonstrated that the genetic causality assessment between hyperthyroidism and RA was not influenced by any individual SNPs (Fig. 3c**)**. Furthermore, the MR-RAPS analysis revealed that the genetic causality assessment between hyperthyroidism and RA followed a normal distribution **(**Table 1; Fig. 3d**)**.

**Table 1:** Sensitivity analysis of the MR analysis results of exposures and outcomes

	Exposure	Outcome	Heterogeneity		Pleiotropy	MR-PRESSO		MR-RAPS
			Cochran’s Q Test (IVW)	Rucker’s Q Test (MR-Egger)	Intercept (MR-Egger)	Outlier	Pleiotropy	Normal Distribution
			*P* value	*P* value	*P* value	Number	*P* value	*P* value
Test cohort	Hyperthyroidism	Rheumatoid arthritis	0.076	0.059	0.578	0	0.116	0.217
	Hypothyroidism		0.145	0.180	0.144	11	0.160	0.397
Validation cohort	Hyperthyroidism	Rheumatoid arthritis	0.011	0.022	0.437	0	0.141	—
	Hypothyroidism		0.502	0.542	0.181	0	0.507	0.385

**Fig. 4:**
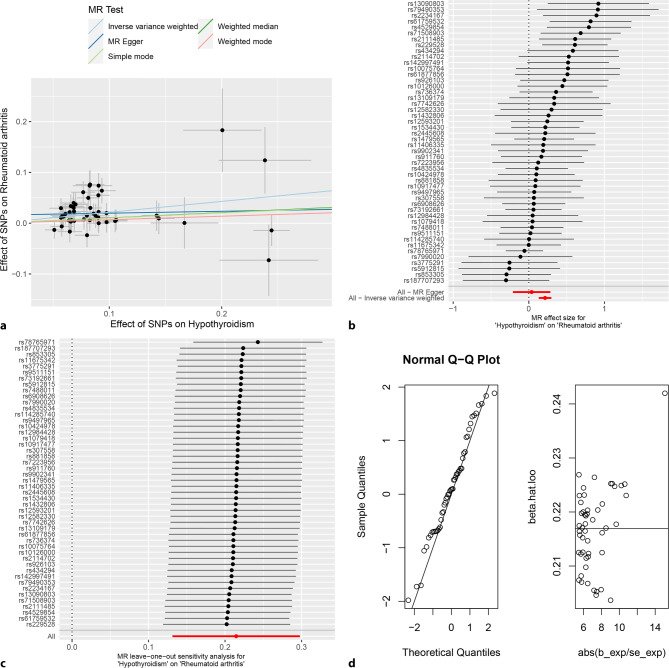
MR analysis of hypothyroidism and rheumatoid arthritis for test cohort. **a** Scatter plot, the effect of SNPs on hypothyroidism in horizontal coordinate and effect of SNPs on rheumatoid arthritis in vertical coordinate. **b** The forest map, contains a total of 48 SNPs. Each horizontal line segment represents an SNP effect estimate and its 95% confidence interval. All-MR-Egger represents a comprehensive estimate of the effect size of all SNPs using the MR-Egger method. All-IVW represents a comprehensive estimate of the effect size of all SNPs using the IVW method. **c** Leave-one-out analysis, delete other SNPs, and use the remaining SNP for MR analysis. All represents an estimate of the total effect size including all SNPs using the IVW method. Each horizontal line represents an estimate of the effect of all SNPs on the result, with the exception of a particular SNP, and its 95% confidence interval. **d** Normal distribution map, to test whether genetic causality conforms to normal distribution. A total of 48 SNPs were included, and the symmetrical distribution of SNPs on both sides of the oblique line indicated that the genetic causality was normally distributed

The random-effects IVW analysis indicated a positive genetic causal relationship between hypothyroidism and RA (*P* < 0.001, OR 95% CI = 1.239 [1.140–1.347]). The other four methods do not yield consistent results (Figs. 2, 4a and 4b**)**. No evidence of heterogeneity or horizontal pleiotropy was found (*P* > 0.05). The 11 outliers were found to be removed during analysis (Table 1**)**. In addition, the “Leave one out” analysis indicated that the genetic causality assessment between hypothyroidism and RA remained consistent even after one remaining SNP (Fig. 4c**)**. The MR-RAPS analysis confirmed that the genetic causality assessment between hypothyroidism and RA followed a normal distribution (*P* > 0.05) (Table 1; Fig. 4d**)**.

### Validation cohort

#### IVs selection

After the exclusion of LD, a total of seven SNPs demonstrated significant correlation with hyperthyroidism, while 56 SNPs exhibited strong correlation with hypothyroidism. From this pool, seven SNPs were selected as IVs for the implementation of MR analysis concerning hyperthyroidism and RA. Notably, two SNPs (rs6679677 and rs9271671) associated with the outcome were excluded from the analysis, as no confounding SNPs were eligible for removal. Furthermore, one SNP displaying incompatible alleles (rs9265890) was eliminated. Consequently, a subset of four SNPs was identified as IVs for the causal assessment of hyperthyroidism and RA, delineated in **Supplementary Table 4**. In the context of the investigation into hypothyroidism and RA, a cohort of 55 SNPs was designated as alternative IVs. Following the exclusion of two SNPs (rs6679677 and rs9273400) linked to the outcome, along with nine confounding SNPs (rs7310615, rs9277542, rs707937, rs10517086, rs11571297, rs11889341, rs244687, rs4409785, rs7902146), and five palindromic SNPs (rs17786733, rs4549506, rs7310615, rs7754251, rs9842232), a set of 40 SNPs was ultimately identified as IVs for the causal evaluation of hypothyroidism and RA, as outlined in **Supplementary Table 5**.

#### MR analysis

**Fig. 5:**
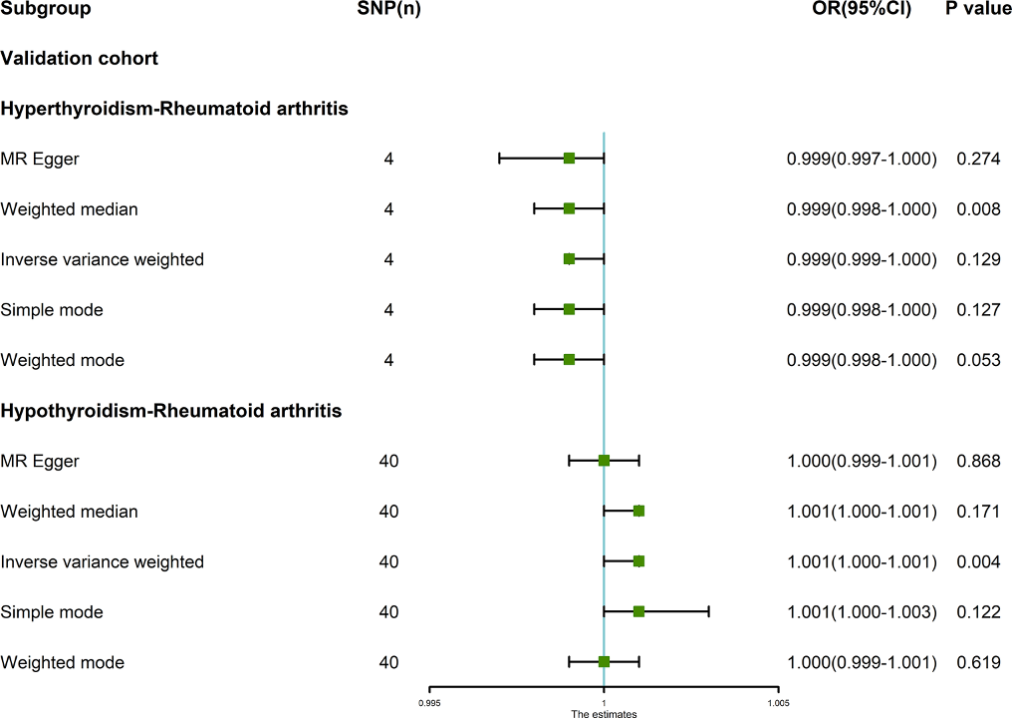
MR analysis results of hyperthyroidism, hypothyroidism and rheumatoid arthritis of validation cohort. Five methods: random-effects IVW, MR Egger, weighted median, simple mode, and weighted mode. The primary analysis method used was random-effects IVW. Random-effects IVW is the gold standard of MR analysis, supplemented by MR Egger, weighted median, simple mode, and weighted mode. If the results of the five methods are inconsistent, the random-effects IVW analysis result prevails

**Fig. 6:**
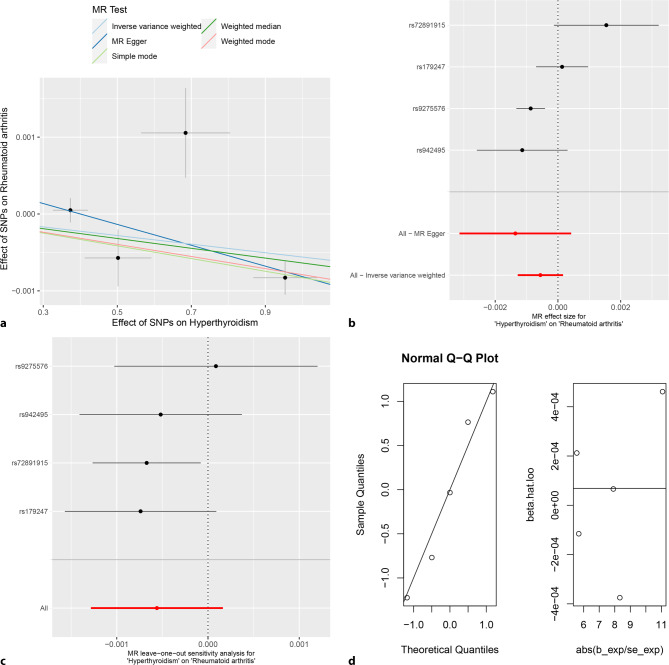
MR analysis of hyperthyroidism and rheumatoid arthritis for validation cohort. **a** Scatter plot, the effect of SNPs on hypothyroidism in horizontal coordinate and effect of SNPs on rheumatoid arthritis in vertical coordinate. **b** The forest map, contains a total of 4 SNPs. Each horizontal line segment represents an SNP effect estimate and its 95% confidence interval. All-MR-Egger represents a comprehensive estimate of the effect size of all SNPs using the MR-Egger method. All-IVW represents a comprehensive estimate of the effect size of all SNPs using the IVW method. **c** Leave-one-out analysis, delete other SNPs, and use the remaining SNP for MR analysis. All represents an estimate of the total effect size including all SNPs using the IVW method. Each horizontal line represents an estimate of the effect of all SNPs on the result, with the exception of a particular SNP, and its 95% confidence interval. **d** Normal distribution map, to test whether genetic causality conforms to normal distribution. A total of 4 SNPs were included, and the symmetrical distribution of SNPs on both sides of the oblique line indicated that the genetic causality was normally distributed

The random-effects IVW analysis revealed no evidence supporting a genetic causal relationship between hyperthyroidism and RA (*P* = 0.129, OR 95% CI = 0.999 [0.999–1.000]). This finding was consistent across various analytical methodologies, including MR Egger, weighted median, simple mode, and weighted mode (*P* > 0.05) (Figs. 5, 6a and 6b**)**. Notably, there exists heterogeneity in the assessment of genetic causality (*P* < 0.05), albeit without evidence of horizontal pleiotropy (*P* > 0.05). Furthermore, no outliers were identified (Table 1**)**. The evaluation of genetic causality between hyperthyroidism and RA remained unaffected by any individual SNPs (Fig. 6c**)**. Moreover, the distribution of genetic causality assessment between hyperthyroidism and RA adhered to a normal distribution pattern (Table 1; Fig. 6d**)**. It is important to highlight that due to the constraints of MR-RAPS, which solely provides *p*-values for MR analyses involving at least seven IVs, the current study, with only four IVs, could not yield a reported *p*-value by MR-RAPS (Table 1**)**.

**Fig. 7:**
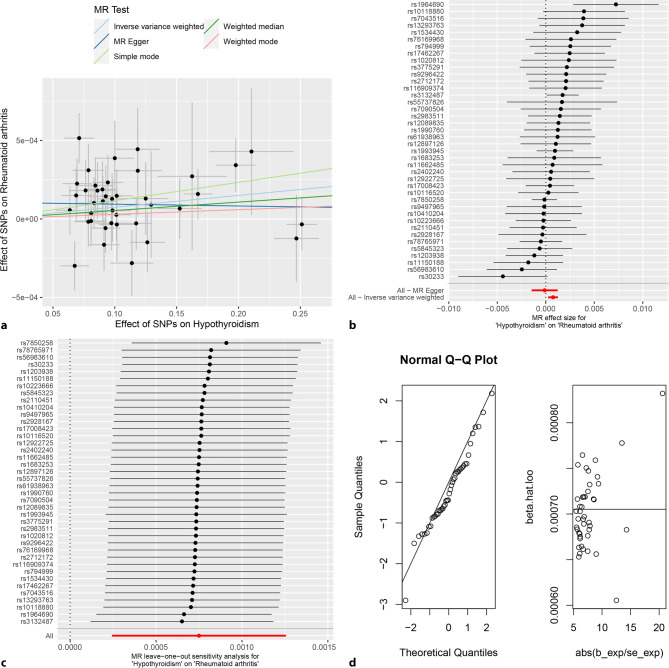
MR analysis of hypothyroidism and rheumatoid arthritis for validation cohort. **a** Scatter plot, the effect of SNPs on hypothyroidism in horizontal coordinate and effect of SNPs on rheumatoid arthritis in vertical coordinate. **b** The forest map, contains a total of 40 SNPs. Each horizontal line segment represents an SNP effect estimate and its 95% confidence interval. All-MR-Egger represents a comprehensive estimate of the effect size of all SNPs using the MR-Egger method. All-IVW represents a comprehensive estimate of the effect size of all SNPs using the IVW method. **c** Leave-one-out analysis, delete other SNPs, and use the remaining SNP for MR analysis. All represents an estimate of the total effect size including all SNPs using the IVW method. Each horizontal line represents an estimate of the effect of all SNPs on the result, with the exception of a particular SNP, and its 95% confidence interval. **d** Normal distribution map, to test whether genetic causality conforms to normal distribution. A total of 40 SNPs were included, and the symmetrical distribution of SNPs on both sides of the oblique line indicated that the genetic causality was normally distributed

The random-effects IVW analysis revealed a positive genetic causal association between hypothyroidism and RA (*P* = 0.004, OR 95% CI = 1.001 [1.000–1.001]). Subsequent MR analyses, encompassing MR Egger, weighted median, simple mode, and weighted mode, yielded findings incongruent with the presence of a genetic causal link between hypothyroidism and RA (*P* > 0.05) (Figs. 5, 7a and 7b**)**. Notably, assessments for heterogeneity, horizontal pleiotropy, and outlier detection did not reveal significant deviations from expected patterns (*P* > 0.05) (Table 1**)**. Further, the “Leave-one-out” analysis indicated that the inferred genetic causality between hypothyroidism and RA remains robust in the retain of individual SNPs (Fig. 7c**)**. Furthermore, the evaluation of genetic causality adhered to a normal distribution (*P* > 0.05) (Table 1; Fig. 7d**)**.

## Discussion

Numerous studies have extensively examined the association between hyperthyroidism, hypothyroidism, and RA. In this study, we employed MR analysis to investigate the causal relationship between hyperthyroidism, hypothyroidism, and RA, focusing on genetic factors. We employed five distinct analytical methodologies in our investigation. Specifically, the random-effects IVW, MR Egger, simple mode, and weighted mode approaches were utilized to explore the potential causal association between hyperthyroidism and RA across both the test and validation cohorts. Results from these methods uniformly suggest the absence of a causal link between hyperthyroidism and RA in both cohorts. Notably, the weighted median analysis reveals no causal relationship in the test cohort but indicates a negative association in the validation cohort. However, it is imperative to underscore that the random-effects IVW method is considered the benchmark in MR analysis. And the sole supplementary approach demonstrating a negative causal connection between hyperthyroidism and RA in the validation cohort. Consequently, the current findings are inconclusive, warranting caution in affirming a causal relationship between hyperthyroidism and RA. Furthermore, employing four supplementary methods, namely MR Egger, weighted median, simple mode, and weighted mode, across both cohorts, our analysis indicates no causal relationship between hypothyroidism and RA. Notably, the random-effects IVW analysis, serving as the gold standard in MR analysis, reveals a positive genetic causality between hypothyroidism and RA in both cohorts. As such, we prioritize the results derived from the random-effects IVW method and conclude that hypothyroidism exhibits a positive genetic causality with RA. The OR values of the hypothyroidism and RA of the test cohort are 1.239, while those of the validation cohort are 1.001. Although the OR values of the analysis results of the two groups of data are different, they both show a positive direction. The result of the test cohort, indicating a 23% increased risk of getting RA for individuals with prevalent hypothyroidism, the validation cohort result showed a 0.10% increased risk of RA with prevalent hypothyroidism. Both sets of results consistently showed an increased risk of rheumatoid arthritis in individuals with hypothyroidism.

Autoimmune thyroid diseases (AITD) encompass both hyperthyroidism and hypothyroidism, constituting the most prevalent organ-specific autoimmune disorders. Graves’ disease predominantly manifests as hyperthyroidism, while Hashimoto’s thyroiditis is primarily associated with hypothyroidism. The prevalence of AITD is approximately 5%, and there is a frequent coexistence of AITD and RA [25]. Certain genetic variants implicated in the regulation of T‑cell responses, such as PTPN22, CTLA 4, and HLA-DR, have been linked to both AITD and RA, indicating a potential shared genetic framework between the two diseases [25]. However, there remains a dearth of conclusive evidence concerning additional associations between AITD and RA, including the causal impact of AITD on the onset of RA and the existence of common environmental triggers. Previous studies investigating the correlation between AITD and RA have predominantly employed a cross-sectional design, with clinical observations often indicating concurrent occurrence in affected individuals. Currently, two prevailing theories attempt to elucidate the temporal sequence of disease manifestation. Some studies have demonstrated an elevated prevalence of RA in patients with AITD [26–28]. Additionally, investigations on episodic RA have suggested that the increased incidence of AITD may predate the diagnosis of RA, signifying that AITD could serve as a preceding factor [29, 30]. Conversely, other reports have indicated a higher prevalence of AITDs in patients with RA [28, 31], or an increased occurrence of thyroid autoantibodies in individuals with RA [32].

RA primarily affects the joints, but it can also involve other systems such as the cardiovascular system, skin, uvea, lungs, and other extra-articular organs. While the exact cause of RA remains incompletely understood, current understanding recognizes the intricate interplay between genetic susceptibility and environmental factors. It is believed that the combined influence of multiple factors contributes to the onset and progression of RA [33]. RA is fundamentally an autoimmune disease, and it is plausible that the etiology of AITD, another autoimmune condition, shares similarities with RA. Although the precise mechanism underlying the shared origins of AITD and RA remains uncertain, both genetic and environmental factors are generally acknowledged as significant contributors to the association between these conditions [34]. Previous investigations have also examined the prevalence of thyroid disease among RA patients in different geographical locations. The prevalence of thyroid disease among RA patients ranged from 0.5% in Morocco to 27% in Slovakia [32, 35]. Similarly, the prevalence of thyroid autoantibodies in RA patients is reported as 5% in the United Kingdom and nearly 30% in Japan [36, 37]. These findings provide compelling evidence for the involvement of genetic susceptibility and environmental factors in the development of autoimmune diseases across diverse populations, supporting the association between AITD and RA.

Both hyperthyroidism and hypothyroidism have been extensively linked to RA. Numerous previous observational studies have consistently reported a stronger correlation between hypothyroidism and RA compared to hyperthyroidism, indicating that hypothyroidism is a more prevalent thyroid dysfunction among RA patients [33]. In this study, we employed MR analysis to investigate the genetic causal relationship between hyperthyroidism, hypothyroidism, and RA at the genetic level. Our findings further confirmed a causal relationship between hypothyroidism and RA, exhibiting a positive genetic causal association. Conversely, no genetic causal relationship was observed between hyperthyroidism and RA. These results align, to some extent, with previous research findings. Notably, hypothyroidism exhibits a stronger association with RA than hyperthyroidism in terms of thyroid dysfunction. We postulate that this disparity may be attributed to the relatively lower immunogenicity of hyperthyroidism compared to hypothyroidism [33]. The majority of hypothyroidism cases stem from autoimmune-mediated Hashimoto’s disease, whereas the prevalence of autoimmune diseases leading to hyperthyroidism ranges from 50 to 80%, depending on factors such as age, geographical region, and iodine intake [38].

There are several limitations to be acknowledged in this study. Firstly, it is important to note that the study population consisted exclusively of individuals from European descent. Given the potential impact of genetic susceptibility and environmental factors, caution should be exercised when generalizing the findings of this study to other populations. Secondly, hyperthyroidism, hypothyroidism, and RA exhibit gender disparities. Although our study did not differentiate between genders due to limitations in the available data, future investigations should consider exploring potential differences in the results when applied specifically to male or female populations.

## Conclusion

This study aimed to investigate the genetic-level causal relationship between hyperthyroidism, hypothyroidism, and RA. The findings revealed that hypothyroidism exhibited a positive causal association with RA, whereas hyperthyroidism did not demonstrate any causal relationship with RA. However, additional investigations are warranted to elucidate their precise connections across different levels in subsequent studies.

## Supplementary Information

Supplementary information provides details of the data used in this study. And details of IVs for which exposure and outcome were evaluated for genetic causality, including SNPs excluded during the IVs screening process. These include SNPs that are associated with outcome, confounding SNPs, and palindromic SNPs.

## Data Availability

Publicly available datasets were analyzed in this study. These data can be found here: IEU OpenGWAS database (https://gwas.mrcieu.ac.uk/).
