# Supplementary material for: A causal relationship between hypothyroidism and rheumatoid arthritis, but not hyperthyroidism: evidence from the mendelian randomization study
Source: Wien Klin Wochenschr. 2024 Jun 20;137(9-10):279–90. doi: 10.1007/s00508-024-02386-6 (PMC12081479; doi:10.1007/s00508-024-02386-6)
Supplement: Supplementary file 1 — Supplementary information provides details of the data used in this study. And details of IVs for which exposure and outcome were evaluated for genetic causality, including SNPs excluded during the IVs screening process. These include SNPs that are associated with outcome, confounding SNPs, and palindromic SNPs. [file 508_2024_2386_MOESM1_ESM.docx]

**Supplementary Table 1:** The GWAS summary data used in this study.

|  |  | **GWAS ID** | **Trait** | **Sample size** | **Number of SNPs** | **Population** |
| --- | --- | --- | --- | --- | --- | --- |
| Test  cohort | exposure | ebi-a-GCST90018860 | Hyperthyroidism | 460,499 | 24,189,279 | European |
|  | exposure | ebi-a-GCST90018862 | Hypothyroidism | 410,141 | 24,138,872 | European |
|  | outcome | finn-b-M13_RHEUMA | Rheumatoid arthritis (M13_RHEUMA) | 153,457 | 16,380,169 | European |
| Validation  cohort | exposure | finn-b-AUTOIMMUNE_HYPERTHYROIDISM | Autoimmune hyperthyroidism | 173,938 | 16,380,189 | European |
|  | exposure | finn-b-E4_HYTHY_AI_STRICT | Hypothyroidism, strict autoimmune | 198,472 | 16,380,353 | European |
|  | outcome | ukb-d-M13_RHEUMA | Rheumatoid arthritis | 361,194 | 10,079,899 | European |

**Supplementary Table 2:** The instrumental variables of MR analysis between hyperthyroidism and rheumatoid arthritis of training cohort.

|  | **SNP** | **beta.exposure** | **se.exposure** | **pval.exposure** | **pval.outcome** | **F** |
| --- | --- | --- | --- | --- | --- | --- |
| 1 | rs1794280 | 0.5463 | 0.0391 | 2.29E-44 | 0.4177 | 195.2122 |
| 2 | rs2160215 | 0.2483 | 0.0226 | 3.82E-28 | 0.09947 | 120.7076 |
| 3 | rs2856821 | -0.1879 | 0.0274 | 7.69E-12 | 7.55E-15 | 47.02735 |
| 4 | rs3087243 | -0.2038 | 0.0224 | 7.94E-20 | 8.55E-10 | 82.77714 |
| 5 | rs385863 | 0.1335 | 0.0214 | 4.09E-10 | 2.70E-05 | 38.91644 |
| 6 | rs4338740 | 0.1844 | 0.0264 | 2.87E-12 | 0.2412 | 48.7879 |
| 7 | rs58722186 | 0.1359 | 0.0233 | 5.23E-09 | 0.9082 | 34.01929 |
| 8 | rs604912 | 0.1198 | 0.0217 | 3.15E-08 | 0.4485 | 30.47841 |
| 9 | rs6131010 | 0.1306 | 0.0237 | 3.60E-08 | 0.01198 | 30.36601 |
| 10 | rs6679677 | 0.2936 | 0.0379 | 8.79E-15 | 1.27E-43 | 60.01113 |
| 11 | rs758778 | 0.3072 | 0.0348 | 1.02E-18 | 0.8335 | 77.92594 |
| 12 | rs9258222 | -0.2367 | 0.0418 | 1.44E-08 | 0.2338 | 32.0658 |

**Supplementary Table 3:** The instrumental variables of MR analysis between hypothyroidism and rheumatoid arthritis of training cohort.

|  | **SNP** | **beta.exposure** | **se.exposure** | **pval.exposure** | **pval.outcome** | **F** |
| --- | --- | --- | --- | --- | --- | --- |
| 1 | rs10075764 | -0.057 | 0.0104 | 4.26E-08 | 0.1415 | 30.03868 |
| 2 | rs10126000 | -0.0683 | 0.0104 | 5.13E-11 | 0.142 | 43.12932 |
| 3 | rs10424978 | -0.0775 | 0.0102 | 2.77E-14 | 0.7006 | 57.72992 |
| 4 | rs1079418 | -0.0657 | 0.011 | 2.14E-09 | 0.8856 | 35.6733 |
| 5 | rs10917477 | 0.064 | 0.01 | 1.75E-10 | 0.776699 | 40.9598 |
| 6 | rs11171710 | -0.0698 | 0.01 | 3.19E-12 | 0.00019 | 48.72016 |
| 7 | rs11406335 | -0.057 | 0.0103 | 3.44E-08 | 0.5866 | 30.62479 |
| 8 | rs114285740 | 0.1669 | 0.0301 | 3.06E-08 | 0.9929 | 30.74522 |
| 9 | rs11675342 | 0.0906 | 0.01 | 1.40E-19 | 0.9948 | 82.0832 |
| 10 | rs11875260 | 0.0751 | 0.0135 | 2.54E-08 | 0.03119 | 30.94641 |
| 11 | rs12117927 | 0.0627 | 0.0105 | 2.29E-09 | 0.0501 | 35.65779 |
| 12 | rs12379417 | 0.0583 | 0.0103 | 1.51E-08 | 0.000112 | 32.03764 |
| 13 | rs12582330 | -0.061 | 0.0109 | 2.05E-08 | 0.3819 | 31.31876 |
| 14 | rs12593201 | 0.0905 | 0.0112 | 7.69E-16 | 0.3214 | 65.29185 |
| 15 | rs12984428 | -0.0659 | 0.0102 | 1.11E-10 | 0.8714 | 41.74153 |
| 16 | rs13090803 | 0.0829 | 0.0128 | 9.00E-11 | 0.007144 | 41.94566 |
| 17 | rs13109179 | 0.0647 | 0.01 | 9.42E-11 | 0.269 | 41.8607 |
| 18 | rs1364450 | 0.0886 | 0.0139 | 1.97E-10 | 0.0641 | 40.62896 |
| 19 | rs142997491 | 0.2385 | 0.0412 | 7.02E-09 | 0.059981 | 33.51045 |
| 20 | rs1432806 | 0.0583 | 0.0105 | 2.89E-08 | 0.4738 | 30.82878 |
| 21 | rs1479565 | 0.0975 | 0.0101 | 7.53E-22 | 0.3154 | 93.18894 |
| 22 | rs1534430 | -0.086 | 0.0101 | 1.44E-17 | 0.3432 | 72.50234 |
| 23 | rs187707293 | 0.2419 | 0.044 | 3.99E-08 | 0.3021 | 30.22486 |
| 24 | rs2111485 | 0.0813 | 0.0102 | 1.43E-15 | 0.0117 | 63.52997 |
| 25 | rs2114702 | 0.07 | 0.0111 | 3.00E-10 | 0.1188 | 39.76931 |
| 26 | rs2234167 | 0.0825 | 0.015 | 3.75E-08 | 0.01348 | 30.24985 |
| 27 | rs2247314 | -0.086 | 0.0104 | 1.06E-16 | 0.000955 | 68.37984 |
| 28 | rs229528 | 0.0903 | 0.01 | 2.31E-19 | 0.005668 | 81.5405 |
| 29 | rs2412976 | 0.0637 | 0.0103 | 5.47E-10 | 0.4476 | 38.24743 |
| 30 | rs2445608 | -0.0593 | 0.0101 | 3.79E-09 | 0.5188 | 34.47184 |
| 31 | rs244685 | -0.0858 | 0.0132 | 7.06E-11 | 0.000295 | 42.24979 |
| 32 | rs2921053 | -0.0599 | 0.0101 | 3.36E-09 | 0.1306 | 35.17295 |
| 33 | rs2988277 | 0.0593 | 0.0106 | 2.49E-08 | 0.001203 | 31.29648 |
| 34 | rs307558 | -0.0688 | 0.0119 | 8.01E-09 | 0.8423 | 33.42573 |
| 35 | rs3087243 | -0.1466 | 0.0102 | 4.77E-47 | 8.55E-10 | 206.5692 |
| 36 | rs3118469 | 0.0803 | 0.0106 | 3.82E-14 | 6.74E-06 | 57.38749 |
| 37 | rs3184504 | -0.1734 | 0.0102 | 7.50E-65 | 5.20E-08 | 288.9986 |
| 38 | rs34536443 | -0.1863 | 0.0263 | 1.46E-12 | 8.34E-08 | 50.17786 |
| 39 | rs3775291 | -0.0649 | 0.0108 | 1.65E-09 | 0.4297 | 36.11102 |
| 40 | rs434294 | -0.0683 | 0.0109 | 3.36E-10 | 0.05811 | 39.26325 |
| 41 | rs4409785 | 0.1069 | 0.0133 | 8.04E-16 | 0.1553 | 64.6026 |
| 42 | rs4529854 | -0.0768 | 0.0107 | 6.56E-13 | 0.005136 | 51.51726 |
| 43 | rs4835534 | -0.1421 | 0.0132 | 7.06E-27 | 0.617701 | 115.8879 |
| 44 | rs5912815 | -0.0511 | 0.0084 | 1.05E-09 | 0.4514 | 37.00676 |
| 45 | rs61759532 | 0.0905 | 0.0122 | 1.42E-13 | 0.003187 | 55.02694 |
| 46 | rs61877856 | -0.0658 | 0.0115 | 1.14E-08 | 0.1483 | 32.73814 |
| 47 | rs6679677 | 0.3637 | 0.0159 | 2.39E-115 | 1.27E-43 | 523.2271 |
| 48 | rs6908626 | 0.1441 | 0.0141 | 2.04E-24 | 0.759299 | 104.445 |
| 49 | rs7030280 | 0.2075 | 0.0108 | 1.02E-82 | 0.9184 | 369.1361 |
| 50 | rs71508903 | 0.0934 | 0.0125 | 9.34E-14 | 0.01103 | 55.83051 |
| 51 | rs7223956 | -0.0902 | 0.0144 | 4.27E-10 | 0.6884 | 39.23611 |
| 52 | rs73192661 | -0.1061 | 0.01 | 4.05E-26 | 0.746 | 112.5716 |
| 53 | rs736374 | 0.0832 | 0.0103 | 6.00E-16 | 0.1423 | 65.24843 |
| 54 | rs7441808 | 0.0766 | 0.0111 | 5.17E-12 | 0.000488 | 47.6222 |
| 55 | rs7488011 | 0.1052 | 0.0111 | 2.52E-21 | 0.843 | 89.82214 |
| 56 | rs7574865 | -0.1321 | 0.0117 | 1.67E-29 | 1.04E-08 | 127.477 |
| 57 | rs7742626 | 0.0686 | 0.0116 | 3.41E-09 | 0.3781 | 34.97278 |
| 58 | rs78765971 | 0.2444 | 0.0162 | 1.68E-51 | 0.644401 | 227.599 |
| 59 | rs79490353 | 0.2006 | 0.0349 | 8.82E-09 | 0.02613 | 33.03763 |
| 60 | rs7990020 | 0.0577 | 0.0101 | 9.97E-09 | 0.7503 | 32.63674 |
| 61 | rs853305 | -0.0802 | 0.0111 | 4.40E-13 | 0.3242 | 52.20362 |
| 62 | rs881858 | 0.0665 | 0.0108 | 8.46E-10 | 0.7755 | 37.91348 |
| 63 | rs911760 | 0.0879 | 0.0125 | 1.95E-12 | 0.5419 | 49.44878 |
| 64 | rs926103 | -0.0678 | 0.0104 | 7.65E-11 | 0.1153 | 42.50016 |
| 65 | rs9264277 | -0.0862 | 0.0111 | 9.03E-15 | 2.60E-18 | 60.30682 |
| 66 | rs9271365 | 0.2484 | 0.0105 | 4.91E-123 | 2.67E-87 | 559.6577 |
| 67 | rs9277559 | -0.133 | 0.012 | 1.86E-28 | 1.57E-20 | 122.8397 |
| 68 | rs9497965 | 0.0827 | 0.0102 | 3.71E-16 | 0.776201 | 65.7368 |
| 69 | rs9511151 | -0.0976 | 0.0106 | 3.08E-20 | 0.9169 | 84.77851 |
| 70 | rs9902341 | 0.0801 | 0.0129 | 4.68E-10 | 0.5364 | 38.55525 |
| Confounding | |  | | | | |
| 1 | rs4409785 | Rheumatoid arthritis | | | | |

**Supplementary Table 4:** The instrumental variables of MR analysis between hyperthyroidism and rheumatoid arthritis of validation cohort.

|  | **SNP** | **beta.exposure** | **se.exposure** | **pval.exposure** | **pval.outcome** | **F** |
| --- | --- | --- | --- | --- | --- | --- |
| 1 | rs179247 | -0.3733 | 0.0472 | 2.47E-15 | 0.755221 | 62.54995 |
| 2 | rs6679677 | 0.4689 | 0.0692 | 1.20E-11 | 3.69E-12 | 45.9138 |
| 3 | rs72891915 | 0.6845 | 0.1204 | 1.31E-08 | 0.070486 | 32.32131 |
| 4 | rs9265890 | 1.1583 | 0.1391 | 8.22E-17 | 0.000448 | 69.33984 |
| 5 | rs9271671 | -0.3124 | 0.0509 | 8.19E-10 | 8.64E-16 | 37.66877 |
| 6 | rs9275576 | 0.9534 | 0.0861 | 1.70E-28 | 0.000191 | 122.6137 |
| 7 | rs942495 | 0.502 | 0.0903 | 2.75E-08 | 0.122603 | 30.90487 |

**Supplementary Table 5:** The instrumental variables of MR analysis between hypothyroidism and rheumatoid arthritis of validation cohort.

|  | **SNP** | **beta.exposure** | **se.exposure** | **pval.exposure** | **pval.outcome** | **F** |
| --- | --- | --- | --- | --- | --- | --- |
| 1 | rs10116520 | 0.1013 | 0.0118 | 7.79E-18 | 0.869289 | 73.69712 |
| 2 | rs10118880 | -0.0787 | 0.0129 | 1.01E-09 | 0.066931 | 37.21908 |
| 3 | rs1020812 | 0.0764 | 0.0135 | 1.72E-08 | 0.342788 | 32.02689 |
| 4 | rs10223666 | 0.1173 | 0.0125 | 7.53E-21 | 0.868313 | 88.05857 |
| 5 | rs10410204 | 0.0786 | 0.0116 | 1.17E-11 | 0.911957 | 45.91184 |
| 6 | rs10517086 | 0.0805 | 0.0128 | 3.29E-10 | 0.010071 | 39.55191 |
| 7 | rs11150188 | -0.0913 | 0.0126 | 3.95E-13 | 0.325115 | 52.50445 |
| 8 | rs11571297 | -0.1303 | 0.012 | 1.20E-27 | 6.61E-05 | 117.9022 |
| 9 | rs11662485 | 0.1294 | 0.0211 | 8.77E-10 | 0.788935 | 37.60965 |
| 10 | rs116909374 | -0.2105 | 0.0341 | 6.56E-10 | 0.285041 | 38.1058 |
| 11 | rs11889341 | 0.1251 | 0.0137 | 7.48E-20 | 0.029484 | 83.38139 |
| 12 | rs1203938 | 0.1263 | 0.0143 | 1.21E-18 | 0.434922 | 78.0064 |
| 13 | rs12089835 | 0.0978 | 0.0124 | 3.55E-15 | 0.433665 | 62.20567 |
| 14 | rs12897126 | -0.125 | 0.0182 | 6.30E-12 | 0.575096 | 47.17076 |
| 15 | rs12922725 | -0.0809 | 0.0132 | 8.21E-10 | 0.843015 | 37.56166 |
| 16 | rs13293763 | 0.1185 | 0.0177 | 2.44E-11 | 0.095179 | 44.82144 |
| 17 | rs1534430 | -0.0695 | 0.0117 | 2.92E-09 | 0.16158 | 35.28528 |
| 18 | rs1683253 | 0.0637 | 0.0116 | 4.50E-08 | 0.721538 | 30.15494 |
| 19 | rs17008423 | -0.1525 | 0.0179 | 1.88E-17 | 0.733721 | 72.58205 |
| 20 | rs17462267 | 0.0944 | 0.0126 | 7.95E-14 | 0.183409 | 56.13045 |
| 21 | rs17786733 | 0.1077 | 0.0117 | 4.13E-20 | 0.685918 | 84.73353 |
| 22 | rs1964690 | -0.0712 | 0.012 | 3.02E-09 | 0.001281 | 35.20409 |
| 23 | rs1990760 | 0.0904 | 0.0117 | 1.22E-14 | 0.482415 | 59.69813 |
| 24 | rs1993945 | 0.1672 | 0.0117 | 4.16E-46 | 0.324004 | 204.2191 |
| 25 | rs2110451 | 0.0972 | 0.0129 | 4.84E-14 | 0.876404 | 56.7739 |
| 26 | rs2402240 | 0.0982 | 0.0152 | 9.21E-11 | 0.786593 | 41.73798 |
| 27 | rs244687 | -0.0767 | 0.0133 | 7.78E-09 | 0.80426 | 33.257 |
| 28 | rs2712172 | -0.09 | 0.012 | 5.55E-14 | 0.291376 | 56.24943 |
| 29 | rs2928167 | -0.1017 | 0.0145 | 2.70E-12 | 0.880709 | 49.1928 |
| 30 | rs2983511 | -0.1016 | 0.0131 | 9.59E-15 | 0.386257 | 60.15067 |
| 31 | rs30233 | -0.0676 | 0.0117 | 8.46E-09 | 0.059488 | 33.38238 |
| 32 | rs3132487 | 0.1978 | 0.0158 | 4.83E-36 | 0.042692 | 156.7235 |
| 33 | rs3775291 | -0.0688 | 0.0124 | 3.31E-08 | 0.381544 | 30.78429 |
| 34 | rs4409785 | 0.0983 | 0.0154 | 1.94E-10 | 0.027789 | 40.74377 |
| 35 | rs4549506 | -0.0661 | 0.0116 | 1.34E-08 | 0.876499 | 32.47002 |
| 36 | rs55737826 | 0.1625 | 0.0279 | 5.91E-09 | 0.564388 | 33.92298 |
| 37 | rs56983610 | -0.1138 | 0.0173 | 4.68E-11 | 0.181083 | 43.2701 |
| 38 | rs5845323 | 0.0925 | 0.0118 | 4.25E-15 | 0.711925 | 61.44904 |
| 39 | rs61938963 | 0.0832 | 0.0124 | 1.68E-11 | 0.539431 | 45.01932 |
| 40 | rs6679677 | 0.3567 | 0.0166 | 1.64E-102 | 3.69E-12 | 461.7274 |
| 41 | rs7043516 | -0.1 | 0.0156 | 1.60E-10 | 0.104746 | 41.09097 |
| 42 | rs707937 | -0.1208 | 0.0146 | 1.27E-16 | 0.01019 | 68.45793 |
| 43 | rs7090504 | 0.0927 | 0.0136 | 8.14E-12 | 0.46077 | 46.45979 |
| 44 | rs7310615 | -0.168 | 0.0117 | 1.50E-46 | 0.025526 | 206.1781 |
| 45 | rs76169968 | -0.1186 | 0.0195 | 1.20E-09 | 0.277947 | 36.99098 |
| 46 | rs7754251 | 0.0883 | 0.0116 | 2.69E-14 | 0.376031 | 57.94301 |
| 47 | rs7850258 | 0.2512 | 0.0122 | 1.44E-93 | 0.836796 | 423.9506 |
| 48 | rs78765971 | 0.2468 | 0.0183 | 2.15E-41 | 0.652299 | 181.8795 |
| 49 | rs7902146 | -0.0818 | 0.0133 | 8.58E-10 | 0.772746 | 37.82674 |
| 50 | rs794999 | -0.0843 | 0.0137 | 8.23E-10 | 0.237451 | 37.86253 |
| 51 | rs9273400 | 0.2572 | 0.0124 | 6.94E-95 | 1.48E-10 | 430.2236 |
| 52 | rs9277542 | -0.1227 | 0.0137 | 3.76E-19 | 0.000679 | 80.21279 |
| 53 | rs9296422 | -0.086 | 0.0144 | 2.36E-09 | 0.319782 | 35.66708 |
| 54 | rs9497965 | 0.0808 | 0.0122 | 4.07E-11 | 0.933688 | 43.86304 |
| 55 | rs9842232 | -0.106 | 0.0118 | 3.17E-19 | 0.191039 | 80.69439 |
| Confounding | |  | | | | |
| 1 | rs7310615 | Ever smoked | | | | |
| 2 | rs9277542 | Rheumatoid arthritis ACPA positive | | | | |
| 3 | rs707937 | Rheumatoid arthritis combined control dataset | | | | |
| 4 | rs10517086 | Rheumatoid arthritis | | | | |
| 5 | rs11571297 | Rheumatoid arthritis | | | | |
| 6 | rs11889341 | Rheumatoid arthritis | | | | |
| 7 | rs244687 | Rheumatoid arthritis | | | | |
| 8 | rs4409785 | Rheumatoid arthritis | | | | |
| 9 | rs7902146 | Rheumatoid arthritis | | | | |
